# Schinzel-Giedion Syndrome with Congenital Megacalycosis in a Turkish Patient: Report of SETBP1 Mutation and Literature Review of the Clinical Features

**DOI:** 10.1155/2017/3740524

**Published:** 2017-12-03

**Authors:** Ozgul Bulut, Zeynep Ince, Umut Altunoglu, Sukran Yildirim, Asuman Coban

**Affiliations:** ^1^Department of Pediatrics, Division of Neonatology, Istanbul University Istanbul Faculty of Medicine, Istanbul, Turkey; ^2^Medical Genetics Department, Istanbul University, Istanbul Faculty of Medicine, Istanbul, Turkey

## Abstract

Schinzel-Giedion syndrome (SGS) is a rare autosomal dominant disorder that results in facial dysmorphism, multiple congenital anomalies, and an increased risk of malignancy. Recently, using exome sequencing, de novo heterozygous mutations in the* SETBP1* gene have been identified in patients with SGS. Most affected individuals do not survive after childhood because of the severity of this disorder. Here, we report* SETBP1* mutation confirmed by molecular analysis in a case of SGS with congenital megacalycosis.

## 1. Introduction

Schinzel-Giedion syndrome (SGS) is a very rare, autosomal dominant syndrome, first described in two siblings in 1978. It is characterized by pattern of severe mental retardation, dysmorphic features, hypertrichosis, and multiple congenital anomalies, including skeletal abnormalities, cardiac, genitourinary, and renal malformations [1, 2]. SGS is caused by de novo germline mutations clustering in a 12 bp hotspot in exon 4 of* SETBP1* [3]. The natural history of this condition includes severe growth retardation and profound developmental delay with visual and hearing defects. Malignant embryonic tumors, particularly neuroepithelial neoplasia, occur in 19% of cases [4]. The outcome is fatal, with half of the affected children dying before the age of 2 years [5].

In this study, we report a case of SGS with congenital megacalycosis with a disease causing mutation. We also present a review of the clinical manifestations of SGS and compare our patient with those reported in the literature.

## 2. Case Report

**Figure 1:**
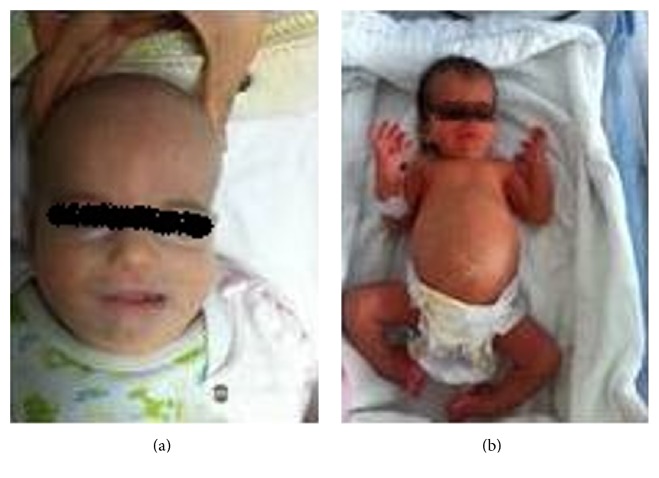
Coarse facial features with midface retraction, frontal bossing, bitemporal narrowing, wide anterior fontanel, low nasal bridge, abdominal distention, and bilateral talipes equinovarus are shown in physical characteristics of the baby.

The proposita was born at 39 1/7 weeks of gestation by spontaneous vaginal delivery after an uneventful pregnancy. The infant was an appropriate size for her gestational age, with a birth weight of 3,020 g. The parents were nonconsanguineous. Antenatal ultrasonography revealed bilateral pelvic dilatation and polyhydramnios. At birth, the patient had coarse facial features with midface retraction, frontal bossing, bitemporal narrowing, wide anterior fontanel, hypertrichosis over the forehead, low nasal bridge, ocular hypertelorism, low set ears, abdominal distention, and bilateral talipes equinovarus (Figure 1).

**Figure 2:**
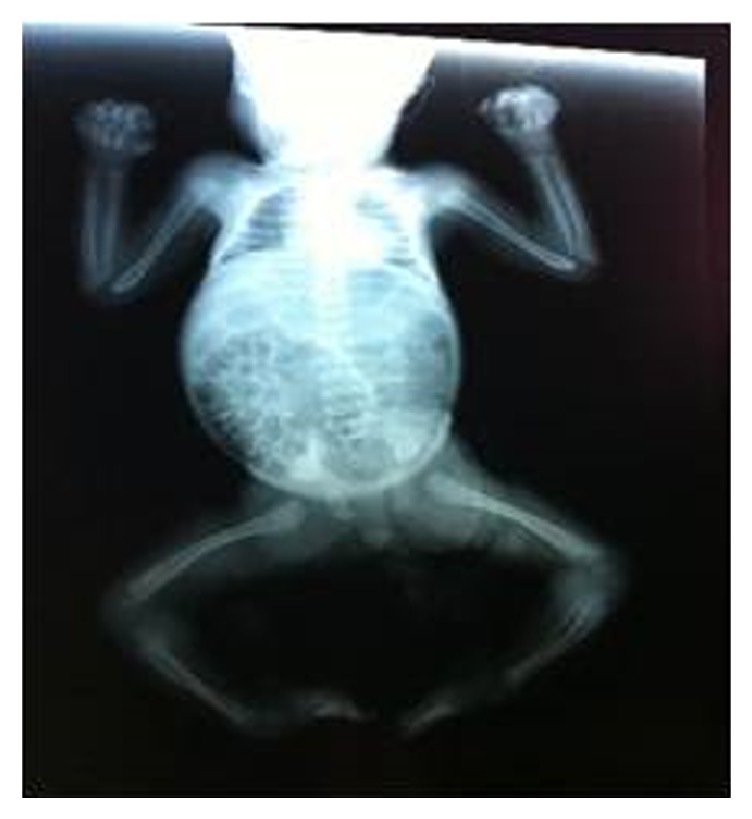
Babygram showing bowed long bones and broad ribs.

Radiological examination showed bowed long bones and broad ribs (Figure 2).

**Figure 3:**
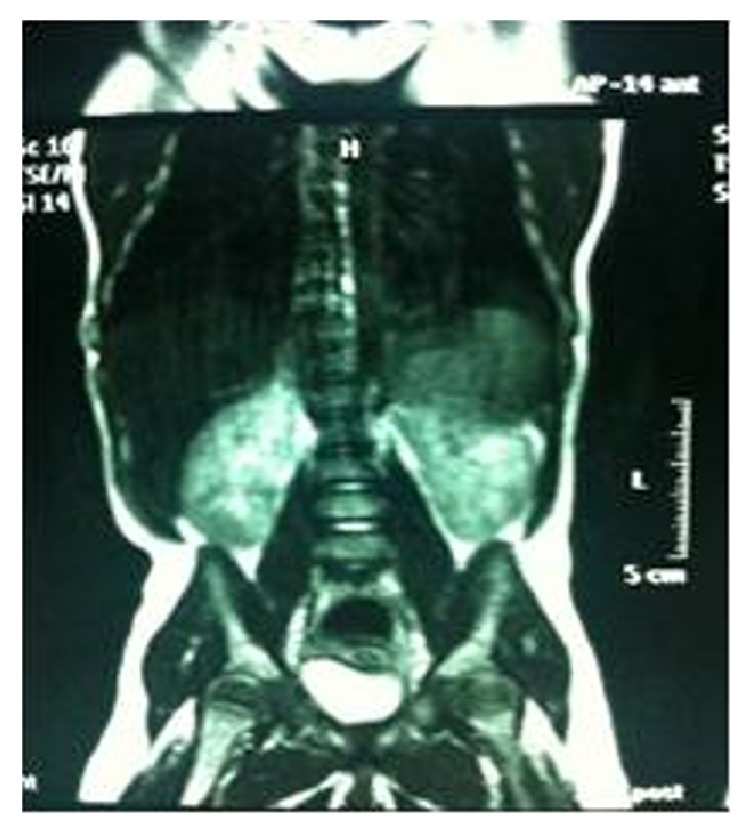
Bilateral megacalycosis with nonobstructive dilation on abdominal MRI.

Echocardiogram revealed patent ductus arteriosus and patent foramen ovale. Abdominal ultrasound showed severe hydronephrosis on the right, moderate hydronephrosis on the left, and diastasis recti. Optic fundus examination and auditory brainstem response testing were normal. Cerebral magnetic resonance imaging (MRI) showed moderate dilatation of the third and lateral ventricles, 2-3 mm choroidal cysts with grossly normal cerebral structures. Bilateral megacalycosis with nonobstructive dilatation was seen on abdominal MRI (Figure 3). Screening tests for inborn metabolism errors were negative. Complete blood count and renal function tests were normal. The patient's karyotype analysis with high resolution banding technique was 46, XX. Sanger sequencing of the* SETBP1* hotspot detected a disease causing heterozygous mutation: c.2608G>A (p.Gly870Ser). On follow-up examination at 8 months of age, the patient had severe growth retardation, developmental delays, feeding problems, and seizures, which were partially controlled with antiepileptic drugs. She had severe visual impairment with absence of fixation and no reaction to bright light. An auditory evoked response test was repeated and showed severe deafness. She died at 11 months of age from respiratory failure.

## 3. Discussion

SGS is a rare developmental disorder characterized by multiple malformations, severe neurological alterations, and an increased risk of malignancy. SGS is caused by de novo germline mutations clustering in a 12 bp hotspot in exon 4 of* SETBP1*. Mutations in this hotspot disrupt a degron, signal regulating protein degradation, and lead to accumulation of the SETBP1 protein [6]. Overlapping somatic mutations in* SETBP1* have been reported in several types of myeloid malignancies [6, 7]. The disease causing mutation in our patient, c.2608G>A (p.Gly870Ser), has been reported previously in the series of Acuna-Hidalgo et al. [8], described in other SGS patients [3, 9], and also as a somatic mutation myeloid malignancies [7].

In SGS, the most important systemic manifestations other than skeletal system malformations are renal defects in the form of hydronephrosis and megacalycosis, cardiac defects, and cerebral ventricular dilatation. Hydronephrosis and various urogenital anomalies are common and may be important in establishing a clinical diagnosis. In 2001, Touge et al. reviewed 35 reported cases and observed hydronephrosis in 31 of them [5]. Hydronephrosis may also be detected during the antenatal period [10]. Other urinary tract abnormalities include hydroureter, double ureter, pyeloureteral junction stenosis, ureterovesical junction stenosis, vesicoureteral reflux, and megacalyces [5]. Congenital megacalycosis has been reported in four patients to date, one of whom was diagnosed prenatally [11–13]. Congenital megacalycosis is believed to be to an abnormal development of the renal medulla leading to hypoplastic renal pyramids and blunted calyces. Megacalycosis may be unilateral or bilateral and is a nonprogressive dilatation of the calyces occasionally accompanied by lithiasis, infection, and hematuria, but with normal renal function. In most cases, the renal pelvis and ureters are not dilated, although primary megaureter can occur with megacalyces [14, 15]. Although usually sporadic, the occurrence of familial megacalyces with autosomal inheritance has supported the possible genetic nature of the malformation [16]. Patients with SGS should undergo detailed renal imaging to determine whether congenital megacalycosis, a common cause of hydronephrosis, is present.

Common neurological problems include severe intellectual disability, intractable epilepsy, and cerebral blindness and deafness. These findings were also seen in our patient during follow-up. One-third of patients with SGS experience intractable neonatal seizures, and another 25% develop West syndrome [17]. Reported nervous system findings comprise a wide spectrum of inconsistent abnormalities, including mild/moderate hydrocephalus, hypoplasia or agenesis of the corpus callosum, cerebral atrophy, absence of cranial nerves with normal cranial nuclei, and abnormal gyration of the cerebral cortex [1, 2, 18, 19].

Patients with SGS usually have trouble swallowing and breathing, also observed in our case. Affected individuals do not survive after childhood, and the most common cause of death is pneumonia. Congenital cardiac defects, tumors, lung hypoplasia, intractable seizures, and sudden cardiac arrest are other causes of death seen during infancy [8].

SGS is an autosomal dominant single-gene disorder, observed sporadically. Prenatal diagnosis can be performed by antenatal ultrasonography to detect polyhydramnios or hydronephrosis and severe skeletal anomalies. In families with history of an affected child, molecular prenatal diagnosis can be offered to rule out the empiric risk of gonadal mosaicism. Genetic counseling for the parents is important for timely prenatal diagnosis.
